# Cytotoxic Oleanane-Type Triterpenoid Saponins from the Rhizomes of *Anemone rivularis* var. *flore-minore*

**DOI:** 10.3390/molecules19022121

**Published:** 2014-02-18

**Authors:** Xiaoyang Wang, Minchang Wang, Min Xu, Yi Wang, Haifeng Tang, Xiaoli Sun

**Affiliations:** 1 Department of Pharmacy, Xijing Hospital, Fourth Military Medical University, Xi’an 710032, China; E-Mail: steveplum@sina.com; 2 Department of Chemistry, School of Pharmacy, Fourth Military Medical University, Xi’an 710032, China; 3 Nuclear Magnetic Resonance Center, Xi’an Modern Chemistry Research Institute, Xi’an 710065, China; E-Mails: wmc204@163.com (M.W.); naturemin2001@163.com (M.X.); 4 Institute of Materia Medica, School of Pharmacy, Fourth Military Medical University, Xi’an 710032, China; E-Mail: yiwang821203@126.com

**Keywords:** *Anemone rivularis* var. *flore-minore*, triterpenoid saponin, cytotoxicity

## Abstract

Phytochemical investigation of the *n*-BuOH extract of the rhizomes of *Anemone rivularis* var. *flore-minore* led to the isolation of five new oleanane-type triterpenoid saponins **1**–**5**, together with five known saponins **6**–**10**. Their structures were determined by the extensive use of 1D and 2D NMR experiments, along with ESIMS analyses and acid hydrolysis. The aglycone of **4** and **5** was determined as 21α-hydroxyoleanolic acid, which was reported in this genus for the first time. The cytotoxicity of these compounds was evaluated against four human cancer cell line, including HL-60 (promyelocytic leukemia), HepG2 (hepatocellular carcinoma), A549 (lung carcinoma) and HeLa (cervical carcinoma). The monodesmosidic saponins **6**–**8** exhibited cytotoxic activity toward all tested cancer cell lines, with IC_50_ values in the 7.25–22.38 μM range.

## 1. Introduction

The genus *Anemone* (Ranunculaceae) consists of about 150 species with a nearly global distribution, of which about 50 species are found in China. More than 10 species of this genus have been used as Chinese folk medicines for a long time. For example, the rhizome of *A. raddeana*, named “Liangtoujian”, is recorded in the Chinese Pharmacopeia for the treatment of rheumatism and neuralgia [1]. Extensive phytochemical and pharmacological studies on this genus have proved the triterpenoid saponins to be the main bioactive substances, with potentially useful biological properties including antitumor, antibacterial, antiperoxidation, insect deterrence, *etc.* [2,3,4,5,6,7,8,9]. *Anemone rivularis* var. *flore-minore* is distributed mainly in the Tsinling Mountains in Shaanxi Province of China. The whole plants of this species are also used as a folk medicine named “Poniuqi” for the treatment of hepatitis, muscle and joint pain, emission, stranguria, edema, *etc.* [10].

**Figure 1:**
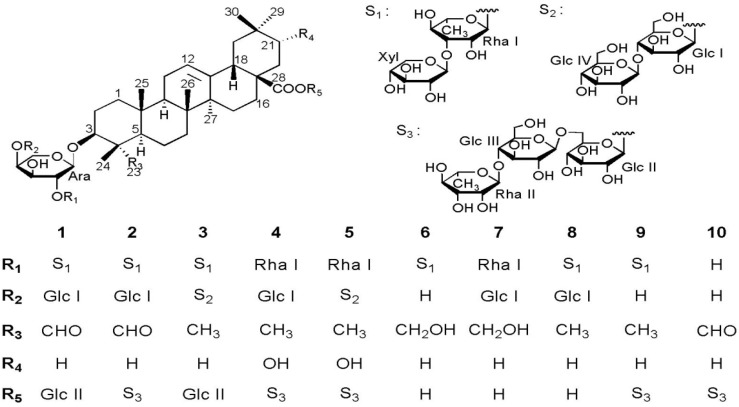
Structures of compounds **1**−**10**.

Our previous phytochemical investigations of the whole plants of this species resulted in the isolation of a new gypsogenin saponin, a new diterpene glycoside, a new lignanoid glycoside, as well as a series of oleanane-type triterpenoid saponins [11,12]. As part of our ongoing search for new bioactive natural compounds from this genus [11,12,13,14,15,16], the present study of the rhizomes of *A. rivularis* var. *flore-minore* led to the isolation of five new oleanane-type saponins **1**−**5**, along with five known saponins **6**−**10** (Figure 1). Herein, we report the isolation and structural elucidation of these saponins, along with their cytotoxic activities against four human cancer cell lines, promyelocytic leukemia HL-60, hepatocellular liver carcinoma HepG2, lung carcinoma A549 and cervical carcinoma HeLa.

## 2. Results and Discussion

Compound **1** was obtained as a white amorphous powder and showed positive results to the Liebermann-Burchard and Molisch tests. The molecular formula C_58_H_92_O_26_ was deduced from the [M+Na]^+^ ion at *m/z* = 1,227.5779 (calcd. for C_58_H_92_O_26_Na^+^, 1,227.5775) in the positive ion mode HRESIMS.

**Table 1:** ^1^H- (500 MHz) and ^13^C-NMR (125 MHz) chemical shifts assignments for the aglycone moieties of compounds **1**−**5** in pyridine-*d*_5_.

C	1		2		3		4		5	
	*δ*_C_	*δ*_H_ (*m*, *J* Hz)	*δ*_C_	*δ*_H_ (*m*, *J* Hz)	*δ*_C_	*δ*_H_ (*m*, *J* Hz)	*δ*_C_	*δ*_H_ (*m*, *J* Hz)	*δ*_C_	*δ*_H_ (*m*, *J* Hz)
1	38.2	0.98, 1.56 m	38.3	1.00, 1.60 m	38.8	1.44, 0.88 m	38.9	0.92, 1.46 m	39.0	0.95, 1.48 m
2	25.5	1.85, 2.09 m	25.5	1.89, 2.09 m	26.7	2.02, 1.81 m	26.7	1.85, 2.06 m	26.7	1.88, 2.10 m
3	81.6	4.05 m	81.7	4.08 m	88.6	3.23 dd (4.0, 11.6)	88.7	3.27 dd (3.9, 11.6)	88.7	3.29 dd (3.8, 11.6)
4	55.5	−	55.7	−	39.5	−	39.5	−	39.5	−
5	47.9	1.36 m	48.0	1.37 m	56.0	0.75 d (11.5)	56.0	0.78 d (11.5)	56.0	0.79 d (11.5)
6	20.6	0.96, 1.38 m	20.6	0.98, 1.42 m	18.5	1.42, 1.24 m	18.5	1.25, 1.43 m	18.5	1.26, 1.47 m
7	32.5	1.18, 1.42 m	32.5	1.19, 1.42 m	33.1	1.40, 1.23 m	33.1	1.24, 1.40 m	33.2	1.25, 1.41 m
8	40.2	−	40.3	−	39.8	−	39.8	−	39.8	−
9	48.2	1.68 m	48.2	1.71 m	48.0	1.60 m	48.1	1.66 m	48.1	1.67 m
10	36.3	−	36.3	−	37.0	−	37.0	−	37.1	−
11	23.4	1.91, 1.99 m	23.5	1.90, 2.02 m	23.7	1.93, 1.86 m	23.7	1.87, 1.93 m	23.7	1.86, 1.94 m
12	122.6	5.40 br s	122.6	5.41 br s	122.8	5.39 br s	122.8	5.43 br s	122.9	5.45 br s
13	144.1	−	144.2	−	144.1	−	144.2	−	144.3	−
14	42.2	−	42.2	−	42.1	−	42.3	−	42.3	−
15	28.3	1.14, 2.03 m	28.3	1.15, 2.06 m	28.3	2.27, 1.14 m	28.5	1.16, 2.30 m	28.6	1.18, 2.30 m
16	23.4	1.76, 2.01 m	23.4	1.77, 2.04 m	23.4	2.05, 1.91 m	27.0	2.36, 3.08 m	27.0	2.33, 3.10 m
17	47.1	−	47.2	−	46.9	−	47.3	−	47.3	−
18	41.7	3.13 dd (3.8, 13.5)	41.7	3.15 dd (3.9, 13.5)	41.6	3.16 dd (3.8, 13.4)	41.7	3.36 dd (3.2, 14.0)	41.8	3.35 dd (3.3, 13.9)
19	46.1	1.23, 1.74 m	46.1	1.23, 1.75 m	46.2	1.71, 1.21 m	41.3	1.71, 1.20 m	41.5	1.75, 1.22 m
20	30.6	−	30.7	−	30.7	−	35.7	−	35.7	−
21	33.9	1.11, 1.34 m	34.0	1.13, 1.36 m	33.9	1.30, 1.08 m	73.3	3.65 br s	73.3	3.66 br s
22	32.5	1.72, 1.89 m	32.5	1.72, 1.90 m	32.5	1.82, 1.74 m	39.6	2.25, 2.28 m	39.7	2.23, 2.29 m
23	206.3	9.61 s	206.4	9.66 s	28.1	1.30 s	28.1	1.29 s	28.2	1.31 s
24	10.5	1.32 s	10.6	1.34 s	17.2	1.15 s	17.2	1.16 s	17.2	1.17 s
25	15.6	0.89 s	15.7	0.89 s	15.5	0.83 s	15.6	0.87 s	15.7	0.88 s
26	17.5	1.05 s	17.5	1.06 s	17.4	1.06 s	17.4	1.09 s	17.4	1.10 s
27	26.2	1.21 s	26.2	1.21 s	26.1	1.23 s	25.6	1.33 s	25.7	1.35 s
28	176.6	−	176.5	−	176.5	−	176.5	−	176.5	−
29	33.1	0.86 s	33.1	0.86 s	33.1	0.86 s	28.3	1.14 s	28.4	1.15 s
30	23.7	0.88 s	23.7	0.87 s	23.7	0.87 s	24.9	1.00 s	25.0	1.01 s

**Figure 2:**
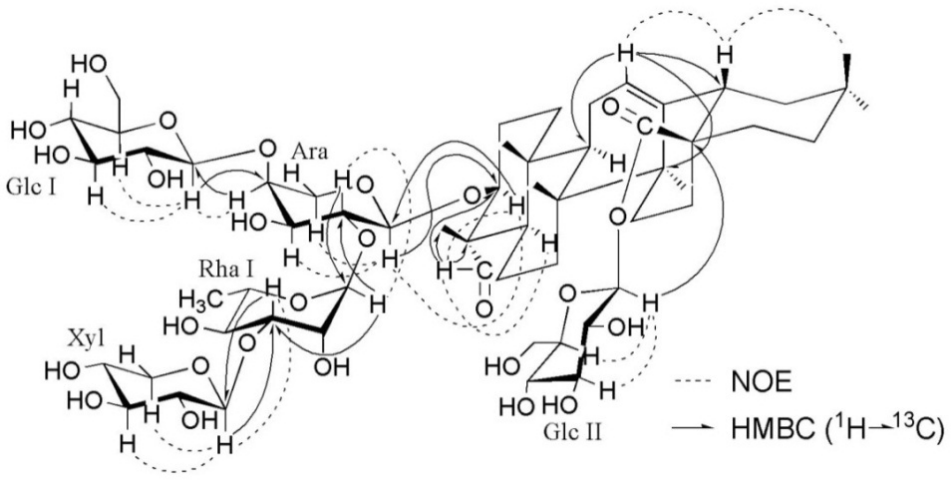
Key NOESY and HMBC correlations for compound **1**.

The analysis of the NMR data (Table 1 and Table 2) indicated that **1** was a saponin containing one triterpene aglycone and five monosaccharides. The 1D-NMR spectra showed signals for six tertiary methyl groups at *δ*_H_ 0.86 (H_3_-29), 0.88 (H_3_-30), 0.89 (H_3_-25), 1.05 (H_3_-25), 1.27 (H_3_-27) and 1.32 (H_3_-24), one olefinic proton signal at *δ*_H_ 5.40 (1H, br s) with two typical olefinic carbon signals at *δ*_C_ 122.6 and 144.1, one aldehyde proton signal at *δ*_H_ 9.61 (1H, s) with the corresponding aldehyde carbon signal at *δ*_C_ 206.3, and one carbonyl signal at *δ*_C_ 176.6, which revealed that the aglycone of **1** was an oleanolic acid derivative with one of the methyl groups substituted by an aldehyde function. The aldehyde function was located at C-23 on the basis of the downfield shift (+16.0 ppm) exhibited by C-4 (*δ*_C_ 55.5) and the highfield shifts (−7.0 ppm, −8.1 ppm, −6.7 ppm) exhibited, respectively, by C-3 (*δ*_C_ 84.6), C-5 (*δ*_C_ 47.9) and C-24 (*δ*_C_ 10.5) in comparison with the same carbon resonances in an oleanene skeleton bearing a Me-23 [16]. The correlations of H-23 (*δ*_H_ 9.61) with H-3 (*δ*_H_ 4.05) and H-5 (*δ*_H_1.36) observed in the NOESY spectrum indicated the α-configuration for the 23-CHO function (Figure 2). The HMBC spectrum of 1 also allowed to determine the position of the aldehyde function by showing the correlations between H-23 (*δ*_H_ 9.61) and C-3 (*δ*_C_ 84.6), C-4 (*δ*_C_ 55.5) and C-24 (*δ*_C_ 10.5) (Figure 2). The assignments of the NMR signals associated with the aglycone moiety were derived from ^1^H-^1^H COSY, TOCSY, HSQC, HMBC and NOESY experiments. These data revealed the aglycone of 1 was gypsogenin [17,18]. The ^13^C-NMR shifts of C-3 at *δ*_C_ 81.6 and C-28 at *δ*_C_ 176.6 implied that sugar linkages were at both C-3 and C-28. The correlations of H-3 with H-23 and H-5 observed in the NOESY spectrum indicated the β-configuration for the 3-*O*-sugar moiety (Figure 2).

**Table 2:** ^1^H- (500MHz) and ^13^C-NMR (125 MHz) chemical shifts assignments for the sugar moieties of compounds **1**−**5** in pyridine-*d*_5_ (m, *J* Hz).

C	1		2		3			4			5	
	*δ*_C_	*δ*_H_	*δ*_C_	*δ*_H_	*δ*_C_	*δ*_H_	*δ*_C_		*δ*_H_	*δ*_C_		*δ*_H_
3-*O*-sugar (Confirm here)												
Ara												
1	104.6	4.92 d (7.1)	104.6	4.91 d (7.2)	105.3	4.72 d (6.9)	105.0		4.77 d (6.2)	105.2		4.74 d (6.2)
2	75.6	4.48 m	75.5	4.50 m	75.5	4.50 m	76.4		4.50 m	76.1		4.49 m
3	75.5	3.91 d (10.3)	75.4	3.90 m	75.0	4.18 m	74.1		4.26 m	74.6		4.25 m
4	80.7	4.10 m	80.8	4.08 m	80.3	4.21 m	79.7		4.27 m	81.0		4.26 m
5	65.8	3.58, 4.35 m	65.9	3.56, 4.32 m	65.3	3.74, 4.38 m	64.5		4.40, 3.80 m	64.9		3.78, 4.41 m
Rha I												
1	101.3	6.33 s	101.2	6.34 s	101.4	6.30 s	101.8		6.15 s	101.7		6.26 s
2	72.0	4.88 br s	71.9	4.87 br s	71.9	4.89 m	72.3		4.71 m	72.2		4.67 m
3	82.9	4.73 m	82.9	4.75 m	82.9	4.72 m	72.6		4.59 m	72.4		4.60 m
4	72.9	4.45 m	73.0	4.47 m	73.0	4.48 m	74.1		4.28 m	74.0		4.25 m
5	69.5	4.71 m	69.6	4.72 m	69.7	4.66 m	69.8		4.62 m	69.7		4.72 m
6	18.5	1.56 d (6.1)	18.5	1.55 d (6.2)	18.5	1.55 d (6.2)	18.6		1.63 d (6.2)	18.6		1.64 d (6.2)
Xyl												
1	107.7	5.34 d (7.6)	107.6	5.35 d (7.7)	107.6	5.34 d (7.7)						
2	75.6	4.03 m	75.5	4.04 m	75.6	4.05 m						
3	78.5	4.13 m	78.5	4.15 m	78.5	4.14 m						
4	71.1	4.12 m	71.0	4.13 m	71.1	4.16 m						
5	67.4	3.68, 4.45 m	67.4	3.68, 4.23 m	67.5	3.73, 4.32 m						
Glc I												
1	106.9	5.08 d (7.9)	106.9	5.08 d (7.9)	106.2	5.03 d (8.0)	106.4		5.13 d (7.9)	106.3		5.04 d (8.0)
2	75.5	3.99 m	75.4	4.01 m	74.9	4.00 m	75.5		4.02 m	74.9		3.99 m
3	78.5	4.18 m	78.5	4.16 m	76.7	4.18 m	78.6		4.19 m	76.7		4.17 m
4	71.2	4.20 m	71.1	4.22 m	81.3	4.29 m	71.3		4.23 m	81.3		4.28 m
5	78.8	3.85 m	78.8	3.86 m	76.7	3.83 m	78.8		3.89 m	76.7		3.82 m
6	62.5	4.33, 4.45 m	62.4	4.36, 4.46 m	61.8	4.41, 4.51 m	62.6		4.37, 4.48 m	61.8		4.40, 4.51 m
Glc IV												
1					105.1	5.15 d (7.8)				105.1		5.15 d (7.8)
2					74.9	4.06 m				74.9		4.05 m
3					78.3	4.19 m				78.3		4.18 m
4					71.6	4.17 m				71.5		4.17 m
5					78.4	3.99 m				78.4		3.98 m
6					62.6	4.26, 4.52 m				62.5		4.27, 4.53 m
28-*O*-sugar												
Glc II												
1	95.6	6.31 d (8.2)	95.6	6.23 d (8.1)	95.7	6.32 d (8.2)	95.6		6.25 d (8.2)	95.7		6.23 d (8.1)
2	74.1	4.19 m	73.9	4.10 m	74.2	4.20 m	73.8		4.08 m	73.8		4.11 m
3	79.3	4.03 m	78.7	4.18 m	79.3	4.02 m	78.7		4.16 m	78.7		4.17 m
4	71.2	4.35 m	70.8	4.28 m	71.2	4.37 m	70.8		4.23 m	70.7		4.29 m
5	78.9	4.27 m	78.0	4.08 m	78.9	4.28 m	78.0		4.06 m	78.0		4.08 m
6	62.2	4.41, 4.44 m	69.2	4.31, 4.64 m	62.3	4.41, 4.46 m	69.1		4.28, 4.62 m	69.0		4.31, 4.63 m
Glc III												
1			104.9	4.97 d (7.7)			104.8		4.97 d (7.8)	104.9		4.98 d (7.8)
2			75.3	3.92 m			75.3		3.93 m	75.3		3.93 m
3			76.5	4.12 m			76.5		4.12 m	76.4		4.14 m
4			78.2	4.40 m			78.2		4.39 m	78.0		4.39 m
5			77.1	3.62 m			77.1		3.64 m	77.1		3.63 m
6			61.2	4.07, 4.18 m			61.2		4.07, 4.17 m	61.1		4.08, 4.18 m
Rha II												
1			102.7	5.84 s			102.7		5.84 s	102.7		5.85 s
2			72.6	4.66 m			72.5		4.65 m	72.6		4.65 m
3			72.7	4.53 m			72.7		4.52 m	72.7		4.53 m
4			74.0	4.31 m			74.0		4.30 m	74.0		4.32 m
5			70.3	4.95 m			70.3		4.94 m	70.2		4.95 m
6			18.5	1.68 d (6.2)			18.5		1.68 d (6.2)	18.5		1.62 d (6.2)

The sugar moieties of **1** were determined to be l-arabinose (Ara), d-xylose (Xyl), l-rhamnose (Rha) and d-glucose (Glc) in a ratio of 1:1:1:2 by acidic hydrolysis followed by comparison of the GC retention times of the corresponding trimethylsilylated hydrolysates with those of the authentic samples prepared in the same manner in the literature [19]. Meanwhile, the ^1^H-NMR spectrum of compound **1** exhibited five anomeric protons at *δ*_H_ = 6.33 (s), 6.31 (d, *J* = 8.2 Hz), 5.34 (d, *J* = 7.6 Hz), 5.08 (d, *J* = 7.9 Hz) and 4.92 (d, *J* = 7.1 Hz), and one methyl group of a 6-deoxyhexopyranosyl moiety at *δ*_H_ 1.56 (d, *J* = 6.1 Hz). The β*-*anomeric configurations for the xylose and glucose units were deduced from their ^3^*J*_H-1/H-2_ coupling constants (7.6−8.2 Hz). The arabinose unit was determined to have an α-anomeric configuration on the basis of the ^3^*J*_H-1/H-2_ (7.1 Hz) value observed in the ^4^*C*_1_ form. Although the anomeric proton of the rhamnose moiety was observed as a singlet in the ^1^H-NMR spectrum, the ^13^C-NMR shift of Rha C-5 at *δ*_C_ = 69.5 indicated the α*-*anomeric configuration [20,21]. All proton signals due to sugars were identified by careful analysis of the ^1^H-^1^H COSY, TOCSY and NOESY spectra, and the carbon signals were assigned by HSQC and further confirmed by HMBC spectra (Table 1). All the monosaccharides were determined to be in their pyranose forms from their ^13^C-NMR data. The sequence and binding sites of the oligosaccharide chains were unambiguously defined by the HMBC experiment. A cross peak between C-3 of the aglycone and H-1 of Ara indicated that Ara was connected to C-3 of the aglycone and a cross peak between C-28 of the aglycone and H-1 of one of the glucose units (Glc II) indicated that Glc II was linked to C-28 of the aglycone.

Similarly, the linkage of one of the glucose units (Glc I) at C-4 of Ara was indicated by the cross peak Glc I H-1/Ara C-4, and the linkages of the terminal xyl at the C-3 of Rha in turn linked to C-2 of Ara were indicated by cross peaks Xyl H-1/Rha C-3 and Rha H-1/Ara C-2. The conclusion was confirmed by the NOESY correlations as shown in Figure 2. On the basis of the above analysis, the structure of compound **1** was thus elucidated as 3-*O*-β-d-xylopyranosyl-(1→3)-α-l-rhamnopyranosyl-(1→2)-[β-d-glucopyranosyl-(1→4)]-α-l-arabinopyranosyl gypsogenin 28-*O*-β-d-glucopyranosyl ester.

Compound **2** was also obtained as a white powder, and showed positive Liebermann-Burchard and Molisch reactions. HRESIMS of **2** showed the quasi-molecular ion at *m*/*z* 1,535.6885 (calcd. for [M+Na]^+^ 1,535.6882), establishing a molecular formula of C_70_H_112_O_35_. The ^1^H- and ^13^C-NMR data assignable to the aglycone moiety of **2** were identical to those of **1** (Table 1), suggesting the aglycone to also be gypsogenin. Seven anomeric protons [*δ*_H_ 6.34 (s), 6.23 (d, *J* = 8.1 Hz), 5.84 (s), 5.35 (d, *J* = 7.7 Hz), 5.08 (d, *J* = 7.9 Hz), 4.97 (d, *J* = 7.7 Hz) and 4.91 (d, *J* = 7.2 Hz)] and seven anomeric carbons (*δ*_C_ 107.6, 106.9, 104.9, 104.6, 102.7, 101.2 and 95.6) were observed in the NMR spectra of **2**. Acid hydrolysis and GC analyses of **2** confirmed the NMR data of the sugar moieties, indicating the units as α-l-arabinose, α-l-rhamnose, β-d-glucose and β-d-xylcose in a 1:2:3:1 ratio (Table 2).

The linkage points of the sugar units to each other and to C-3 and C-28 of the aglycone were established from the HMBC correlations of signals at *δ*_H_ 4.91 (H-1 of Ara) with *δ*_C_ 81.7 (C-3 of the aglycone), *δ*_H_ 6.34 (H-1 of Rha I) with *δ*_C_ 75.5 (C-2 of Ara), *δ*_H_ 5.35 (H-1 of Xyl ) with *δ*_C_ 82.9 (C-3 of Rha I), *δ*_H_ 5.08 (H-1 of Glc I) with *δ*_C_ 80.8 (C-4 of Ara), *δ*_H_ 6.23 (H-1 of Glc II) with *δ*_C_ 176.5 (C-28 of the aglycone), *δ*_H_ 4.97 (H-1 of Glc III) with *δ*_C_ 69.2 (C-6 of Glc II) and *δ*_H_ 5.84 (H-1 of Rha II) with *δ*_C_ 78.2 (C-4 of Glc III) (Figure S1 in the Supplementary Data). Accordingly, compound **2** was identified as 3-*O*-β-d-xylopyranosyl-(1→3)-α-l-rhamnopyranosyl-(1→2)-[β-d-glucopyranosyl-(1→4)]-α-l-arabino-pyranosyl gypsogenin 28-*O*-α-l-rhamnopyranosyl-(1→4)-β-d-glucopyranosyl-(1→6)-β-d-glucopyranosyl ester.

Compound **3** was positive to Liebermann-Burchard and Molisch tests. The molecular formula was established as C_64_H_104_O_30_ from the [M+Na]^+^ ion at *m/z* = 1,375.6507 (calcd. for C_70_H_114_O_34_Na^+^, 1,375.6510) in the positive ion mode HRESIMS. The analysis of the NMR data (Table 1 and Table 2) indicated that **3** was a saponin containing one triterpene aglycone and six monosaccharides. The aglycone of **3** was recognized to be oleanolic acid by ^1^H- and ^13^C-NMR analysis (Table 1), which was in full agreement with literature data [21]. The chemical shifts of C-3 (*δ*_C_ = 88.6) and C-28 (*δ*_C_ = 176.5) revealed that **3** was a bisdesmosidic glycoside. The presence of l-arabinose (Ara), d-xylose (Xyl), l-rhamnose (Rha) and d-glucose (Glc) in a 1:1:2:2 ratio was established by acid hydrolysis followed by GC analysis of the corresponding derivatives. Complete ^1^H- and ^13^C-NMR assignments of the sugar part were achieved by a combination of 2D-NMR experiments (Table 2). The NMR data of the sugar part of **3** were very similar to those obtained from **1**, except for a significant downfield shift of C-4 (*δ*_C_ = 81.3) of Glc I and the appearance of a set of additional signals, corresponding to a terminal β-d-glucopyranosyl group (Glc IV) in **3** which was attached at C-4 of Glc I. The sequence and binding sites of the sugar units connected to C-3 and C-28 of the aglycone were confirmed by the HMBC and NOESY correlations (Figure S2 in Supplementary Data). Hence, compound **3** was established as 3-*O*-β-d-xylopyranosyl-(1→3)-α-l-rhamnopyranosyl-(1→2)-[β-d-glucopyranosyl-(1→4)-β-d-gluco-pyranosyl-(1→4)]-α-l-arabinopyranosyl oleanolic acid 28-*O*-β-d-glucopyranosyl ester.

Compound **4** was obtained as white amorphous powder and showed positive results to the Liebermann-Burchard and Molisch tests. In the positive-ion mode HRESIMS, a pseudomolecular ion peak at *m/z* 1,405.6612 [M+Na]^+^ (calcd. for C_65_H_106_O_31_Na^+^, 1,405.6616) was observed, which is consistent with a molecular formula C_65_H_106_O_31_. The 1D-NMR data of **4** exhibited signals for seven tertiary methyl groups at *δ*_H_ 0.87 (H_3_-25), 1.00 (H_3_-30), 1.09 (H_3_-26), 1.14 (H_3_-29), 1.16 (H_3_-24), 1.29 (H_3_-23) and 1.33 (H_3_-27), one olefinic proton at *δ*_H_ 5.43 (1H, br s) with two typical olefinic carbon signals at *δ*_C_ 122.8 and 144.2, and one carbonyl signal at *δ*_C_ 176.5, which revealed that the aglycone of **4** was a derivative of oleanolic acid. Comparison of their chemical shifts between the aglycone and oleanolic acid indicated that they were the same except for the E-ring. Due to the change of chemical shift of C-21 from *δ*_C_ 31.4 in oleanolic acid [22] to *δ*_C_ 73.3 (+41.9) and the other of carbons such as C-18 [*δ*_C_ 41.7 (−1.2)], C-19 [*δ*_C_ 41.3 (−5.0)], C-20 [*δ*_C_ 35.7 (+4.8)], C-22 [*δ*_C_ 39.6 (+6.4)], C-29 [*δ*_C_ 28.3 (−6.9)] and C-30 [*δ*_C_ 24.9 (+0.2)], C-21 must be an oxygen-bearing methylene carbon in the aglycone of **4**, which was confirmed by the DEPT experiment. In the HMBC experiment, the correlation peaks H_3_-29/C-21 and H_3_-30/C-21 also allowed the location of a hydroxyl group at C-21 (Figure S3 in Supplementary data). The NOESY correlations between H-21 (*δ*_H_ 3.65) and H_3_-30 (*δ*_H_ 1.00) indicated the α-orientation of 21-OH. The assignments of the NMR signals associated with the aglycone moiety were derived from ^1^H-^1^H COSY, TOCSY, HSQC, HMBC and NOESY experiments. These data revealed the aglycone of **4** was 21α-hydroxy-oleanolic acid, which was in a good agreement with the literature data [23,24]. The ^1^H- and ^13^C-NMR spectra assignable to the sugar moieties of **4** were similar to those of **2** except for the absence of proton and carbon resonances for a terminal β-d-xylose moiety. The sequence and binding sites of the sugar units to each other and to the aglycone were confirmed by the HMBC and NOESY correlations (Figure S3 in the Supplementary Data). On the basis of the above analysis, the structure of compound **4** was elucidated as 3-*O*-α-l-rhamnopyranosyl-(1→2)-[β-d-glucopyranosyl-(1→4)]-α-l-arabinopyranosyl 21α-hydroxyoleanolic acid 28-*O*-α-l-rhamnopyranosyl-(1→4)-β-d-glucopyranosyl-(1→6)-β-d-glucopyranosyl ester.

Compound **5** was also obtained as a white powder, showed positive Libermann-burchard and Molisch reactions. HRESIMS of **5** showed the quasi-molecular ion at *m*/*z* 1,567.7150 (calcd. for [M+Na]^+^ 1,567.7144), establishing the molecular formula of C_71_H_116_O_36_. The 1D-NMR data assignable to the aglycone moiety of **5** were identical to those of **4** (Table 1), suggesting the aglycone also to be 21α-hydroxy-oleanolic acid. The ^1^H- and ^13^C-NMR spectra assignable to the sugar moieties of **5** were similar to those of **4** except for the presence of proton and carbon resonances for an additional β-d-glucopyranose moiety (Glc IV). The downfield-shifted carbon signal of Glc I C-4 (*δ*_C_ 81.3) in the ^13^C-NMR spectrum and the presence of a correlation between Glc IV H-1 (*δ*_H_ 5.15) and Glc I C-4 (*δ*_C_ 5.15) in the HMBC spectrum indicated that Glc IV was attached to Glc I C-4. The conclusion was confirmed by the NOESY correlations (Figure S4 in the Supplementary Data). Hence, compound **5** was established as 3-*O*-β-d-glucopyranosyl-(1→4)-β-d-glucopyranosyl-(1→4)-[α-l-rhamno-pyranosyl-(1→2)]-α-l-arabinopyranosyl 21α-hydroxyoleanolic acid 28-*O*-α-l-rhamnopyranosyl-(1→4)-β-d-glucopyranosyl-(1→6)-β-d-glucopyranosyl ester.

By comparing their physical and spectroscopic data with those reported in the literature, the known compounds were identified as sapindoside B (**6**) [25], pulsatilla saponin D (**7**) [26], 3-*O*-β-d-xylopyranosyl-(1→3)-α-l-rhamnopyranosyl-(1→2)-[β-d-glucopyranosyl-(1→4)]-α-l-arabinopyranosyl oleanolic acid (**8**) [27], sieboldianoside B (**9**) [28], and 3-*O*-α-l-arabinopyranosyl gypsogenin 28-*O*-α-l-rhamnopyranosyl-(1→4)-β-d-glucopyranosyl-(1→6)-β-d-glucopyranosyl ester (**10**) [11], respectively.

The cytotoxic activity of saponins **1**−**10** against human leukemia HL-60 cells, human hepatocellular carcinoma HepG2 cells, human lung carcinoma A549 cells and human cervical carcinoma HeLa cells were evaluated by MTT colorimetric assay. Doxorubicin was used as positive control.

The IC_50_ value of each compound was measured on the basis of cell viability after 72 h treatment. As shown in Table 3, all monodesmosidic saponins **6**−**8**, which possess oligosaccharide chains at C-3 and a free carboxylic acid at C-28 of the aglycone, exhibited cytotoxic activity against all tested cancer cell lines with IC_50_ values ranging from 7.25 to 22.38 μM. The bisdesmosidic saponins **2**, **4**, **5**, **9** and **10**, which possess the same α-l-rhamnopyranosyl-(1→4)-β-d-glucopyranosyl-(1→6)-β-d-gluco-pyranosyl oligosaccharide chain at C-28, were all inactive. Earlier studies on the cytotoxicity of similar compounds have reached the same results [29,30].

**Table 3:** Cytotoxic activity of compounds **1**−**10** against four human cancer cell lines *in vitro*.

Compounds ^a^	Cytotoxic activity (IC_50_, μM; mean ± SD, *n* = 3)			
	HL-60	HepG2	A549	HeLa
**1**	82.16 ± 2.79	>100	>100	>100
**3**	45.32 ± 1.74	55.79 ± 2.57	75.68 ± 2.79	>100
**6**	12.32 ± 0.41	13.25 ± 0.38	19.42 ± 0.67	22.20 ± 0.42
**7**	9.57 ± 0.54	10.64 ± 0.47	22.38 ± 0.72	17.31 ± 0.81
**8**	7.25 ± 0.31	12.11 ± 0.87	9.89 ± 0.57	15.47 ± 0.73
Doxorubicin ^b^	0.35 ± 0.05	0.50 ± 0.04	0.68 ± 0.10	0.66 ± 0.07

HL-60—humanpromyelocyticleukemia cell line; HepG2—human hepatocellular carcinoma cell line; A549—human lung carcinoma cell line; HeLa—human cervical carcinoma cell line; ^a^ Compounds **2**, **4**, **5**, **9** and **10** were inactive (IC_50_ > 100 μM) for all cell lines; ^b^ Doxorubicin was used as positive control.

Interestingly, the remaining two bisdesmosidic saponins **1** and **3** showed moderate cytotoxicity (**1** against HL-60; **3** against HL-60, HeLa and A549). This may be ascribed to the fact that these two saponins only possess one sugar moiety at C-28 of the aglycone. Therefore, we postulated that the shorter oligosaccharide chain at C-28 the better cytotoxic activity the saponins would exhibit.

## 3. Experimental

### 3.1. General

Optical rotations were measured on a Perkin-Elmer 343 polarimeter. The ESIMS and HRESIMS were measured on a Micromass Quattro mass spectrometer. NMR experiments were performed on a Bruker AVANCE-500 spectrometer in pyridine-*d_5_* (99.95%, Sigma-Aldrich) with TMS as internal standard. GC was performed on a Finnigan Voyager apparatus using an L-Chirasil-Val column (0.32 mm × 25 m; injector temperature: 230 °C; column temperature: 100−180 °C, rate 5 °C/min; column head pressure: 12 Pa; carrier gas: He, 2 mL/min). HPLC was carried out on a Dionex P680 liquid chromatograph equipped with a UV 170 U*V/V* is detector at 206 nm using a Diamonsil C18(2) column (21.2 × 250 mm i.d., 5 μm, Dikma Technologies Inc., Lake Forest, CA, USA). Materials for column chromatography (CC) were silica gel (10−40 μm, Qingdao Marine Chemical Inc., Qingdao, China), Sephadex LH-20 (40−70 μm, GE-Healthcare, Uppsala, Sweden), and reversed phase silica gel ODS-A (50 μm, YMC Co., Ltd, Kyoto, Japan). TLC detection was achieved by spraying the silica gel plates (Qingdao Marine Chemical Inc) with 20% H_2_SO_4_-EtOH (*v/v*) solution followed by heating. The Liebermann–Burchardtest was made with acetic anhydride and sulfuric acid, and the Molisch test was made with α-naphthol and sulfuric acid. Chemical reagents for isolation were of analytical grade and purchased from Tianjin Fuyu Chemical Co. Ltd. (Tianjin, China).

### 3.2. Plant Material

The rhizomes of *Anemone rivularis* var. *flore-minore* were collected on Taibai Mountain, Shaanxi Province, China, in September 2012, and identified by Prof. Ji-Tao Wang (Department of Pharmacognosy, School of Pharmacy, Shaanxi University of Chinese Medicine). A voucher specimen (NO.120922) has been deposited in the Herbarium of Shaanxi University of Chinese Medicine.

### 3.3. Extraction and Isolation

The air-dried rhizomes of *A. rivularis* var. *flore-minore* (8 kg) were powdered and extracted with 70% EtOH (16 L) under reflux for three times (each for 2 h). The extract was evaporated *in vacuo* to yield a residue (1,300 g) which was suspended in water (10 L) and partitioned successively with petroleum ether (10 L × 2) and *n*-BuOH (10 L × 2). The *n*-BuOH extract (160 g) was separated by silica gel CC using a stepwise gradient of CHCl_3_-MeOH-H_2_O (10:1:0.05−6:4:0.8) to give nine fractions (Fr. 1−Fr. 9). Fr. 3 (4.5 g) was subject to silica gel CC with a CHCl_3_–MeOH–H_2_O gradient (10:1:0.1−7:3:0.5) to give five sub-fractions (Fr. 3.1−Fr. 3.5). Fr. 3.2 (0.8 g) and Fr. 3.3 (1.2 g) were submitted to gel permeation chromatography on Sephadex LH-20 in MeOH to remove the pigments and carbohydrates, and further purified by semipreparative HPLC to give compound **6** [35 mg, MeOH–H_2_O (75:25), 8 mL/min, *t*_R_ 22.0 min] and compound **7** [42 mg, MeOH–H_2_O (78:22), 8 mL/min, *t*_R_ 19.5 min], respectively. Fr. 4 (3.5 g) was separated by silica gel CC with a stepwise gradient of CHCl_3_–MeOH–H_2_O gradient (10:1:0.1−7:3:0.5) to yield three fractions (Fr. 4.1−Fr. 4.3). Compound **8** (26 mg) was obtained from Fr. 4.2 by semipreparative HPLC [MeOH-H_2_O (82:18), 8 mL/min, *t*_R_ 16.5 min]. Fr. 6 (2.1 g) and Fr. 7 (15.5 g) were subjected to ODS CC using a stepwise gradient [MeOH–H_2_O (1:4−10:1)] to afford Fr. 6.1−Fr. 6.5 and Fr. 7.1−Fr. 7.6, respectively. Fr. 6.2 (0.9 g) were submitted to Sephadex LH-20 CC in MeOH and further purified by semipreparative HPLC [MeOH–H_2_O (75:25), 8 mL/min) to yield compound **9** (35 mg, *t*_R_ 19.7 min) and compound **3** (26 mg, *t*_R_ 25.2 min). Fr. 7.2 (1.1 g) and Fr. 7.3 (1.5 g) were subjected to semipreparative HPLC after CC over Sephadex LH-20 (MeOH), to give compound **10** [45 mg, MeOH-H_2_O (62:38), 6.5 mL/min, *t*_R_ 18.5 min from Fr. 7.2], compound **1** [24 mg, MeOH–H_2_O (55:45), 6.0 mL/min, *t*_R_ 29.3 min from Fr. 7.3] and compound **2** [30 mg, MeOH-H_2_O (55:45), 6.0 mL/min, *t*_R_ 35.8 min from Fr. 7.3]. Fr. 7.4 (8.6 g) was separated by ODS CC again eluting with a gradient of MeOH–H_2_O (1:10−5:1) to afford Fr. 7.4.1−Fr. 7.4.6. Compound **4** [15 mg, MeOH–H_2_O (70:30), 8 mL/min, *t*_R_ 18.5 min] and Compound **5** (19 mg, MeOH-H_2_O (65:35), 7 mL/min, *t*_R_ 25.6 min) were obtained from Fr. 7.4.2 and 7.4.3, respectively. Purities of these compounds were determined >95% by HPLC.

Compound **1**: White amorphous powder; [α]
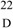
+12.3 (*c* 0.16, MeOH); ^1^H- and ^13^C-NMR data, see Table 1 and Table 2; key HMBC and NOESY correlations, see Figure 2; ESIMS (pos. ion mode) *m/z* 1227 [M+Na]^+^; ESIMS (neg. ion mode) *m/z* 1203 [M−H]^−^, 1071 [1203−132]^−^, 1041 [1203−162]^−^, 925 [1071−146]^−^, 909 [1071−162]^−^, 879 [1041−162]^−^; HRESIMS (pos. ion mode) *m/z* 1227.5779 [M+Na]^+^ (calcd. for C_58_H_92_NaO_26_, 1227.5775).

Compound **2**: White amorphous powder; [α]
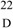
−8.2 (*c* 0.18, MeOH); ^1^H- and ^13^C-NMR data, see Table 1 and Table 2; key HMBC and NOESY correlations, see Figure S1 in the Supplementary Data; ESIMS (pos. ion mode) *m/z* 1535 [M+Na]^+^, 1065 [1535−146−162−162]^+^; ESIMS (neg. ion mode) *m/z* 1511 [M−H]^−^, 1379 [1511−132]^−^, 1349 [1511−162]^−^, 1041 [1511−146−162−162]^−^; HRESIMS (pos. ion mode) *m*/*z* 1535.6885 [M+Na]^+^ (calcd. for C_70_H_112_NaO_35_, 1535.6882).

Compound **3**: White amorphous powder; [α]
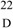
−15.4 (*c* 0.20, MeOH); ^1^H- and ^13^C-NMR data, see Table 1 and Table 2; key HMBC and NOESY correlations, see Figure S2 in the Supplementary Data; ESIMS (pos. ion mode) *m/z* 1375 [M+Na]^+^, 1243 [1375−132]^+^, 1213 [1375−162]^+^, 1081 [1243−162]^+^, 1051 [1213−162]^+^; ESIMS (neg. ion mode) *m/z* 1351 [M−H]^−^, 1219 [1351−132]^−^, 1189 [1351−162]^−^, 1027 [1189−162]^−^; HRESIMS (pos. ion mode) *m/z* 1375.6507 [M+Na]^+^ (calcd. for C_70_H_114_NaO_34_, 1375.6510).

Compound **4**: White amorphous powder; [α]
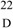
+7.2 (*c* 0.11, MeOH); ^1^H- and ^13^C-NMR data, see Table 1 and Table 2; key HMBC and NOESY correlations, see Figure S3 in the Supplementary Data; ESIMS (pos. ion mode) *m/z* 1405 [M+Na]^+^, 1259 [1405−146]^+^, 1243 [1405−162]^+^, 935 [1405−146−162−162]^+^; ESIMS (neg. ion mode) *m/z* 1381 [M−H]^−^, 911 [1381−146−162−162]^−^; HRESIMS (pos. ion mode) *m/z* 1405.6612 [M+Na]^+^ (calcd. for C_65_H_106_NaO_31_, 1405.6616).

Compound **5**: White amorphous powder; [α]
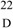
−9.4 (*c* 0.18, MeOH); ^1^H- and ^13^C-NMR data, see Table 1 and Table 2; key HMBC and NOESY correlations, see Figure S4 in the Supplementary Data; ESIMS (pos. ion mode) *m/z* 1567 [M+Na]^+^, 1421 [1567−146]^+^, 1405 [1567−162]^+^, 1097 [1567−146−162−162]^+^; ESIMS (neg. ion mode) *m/z* 1543 [M−H]^−^, 1073 [1543−146−162−162]^−^; HRESIMS (pos. ion mode) *m*/*z* 1567.7150 [M+Na]^+^ (calcd. for C_71_H_116_NaO_36_,1567.7144).

### 3.4. Acid Hydrolysis and GC Analysis of the Sugar Moieties in **1**–**5**

Compounds **1**−**5** (4 mg each) in 1 M HCl (dioxane–H_2_O 1:1, *v/v*, 5 mL) were heated at 95 °C for 6 h, respectively. The reaction mixture was evaporated *in vacuo* and the residue was extracted three times with CHCl_3_. The aqueous phase was concentrated and dissolved in pyridine (5 mL) and 1-(trimethylsilyl)imidazole (0.5 mL) at room temperature for 30 min. The reaction mixture was dried with a stream of N_2_. The residue was partitioned between CHCl_3_ and H_2_O. The organic layer was subjected to GC analysis using an L-Chirasil-Val column. The sugar units were identified by comparing the retention times of the corresponding trimethylsilylated derivatives with those of the authentic samples prepared in the same manner [19]. Retention times for authentic samples after being derivatized were 9.12 and 10.05 min (d-arabinose), 9.30 and 10.29 min (l-arabinose), 9.90 and 10.69 min (d-rhamnose), 9.70 and 10.58 min (l-rhamnose), 11.15 and 12.36 min (d-xylose), 11.27 and 12.30 min (l-xylose), 14.95 min (d-glucose), and 15.18 min (l-glucose), respectively. l-Arabinose, l-rhamnose, d-xylose and d-glucose were identified in a ratio of 1:1:1:2 for **1**, 1:2:1:3 for **2** and 1:2:1:4 for **3**, while the sugar moieties of **4** and **5** were identified as l-arabinose, l-rhamnose and d-glucose in the ratio of 1:2:3 and 1:2:4, respectively.

### 3.5. Assays for *in Vitro* Antitumor Activity

The cytotoxicity of compounds **1**−**5** against human promyelocytic leukemia HL-60 cells, human hepatocellular carcinoma HepG2 cells, human lung carcinoma A549 cells and human cervical carcinoma HeLa cells (all four cancer cell lines were obtained from ATCC, Manassas, VA, USA) was evaluated by MTT colorimetric assay described in previous papers [31], with doxorubicin (Sigma-Aldrich, St. Louis, MO, USA) as positive control. Briefly, 4 × 10^3^/mL cells were added to 96-well plates (100 μL/well), and incubated with various concentrations of drugs (80, 40, 20, 10, 5, 1 and 0.2 μM) in triplex wells for 48 h at 37 °C in a humidified 5% CO_2_ atmosphere. After 48 h, 20 μL 3-(4,5-dimethylthiazol-2-yl)-2,5-diphenyltetrazolium bromide (MTT) solved in PBS was added to each well at a concentration of 5 mg/mL, and then incubated for another 4 h. The water-insoluble dark blue formazan crystals formed during MTT cleavage in actively metabolizing cells were dissolved in DMSO. The optical density of each well was measured with a Bio-Rad 680 microplate reader (Bio-Rad, Hercules, CA, USA) at 570 nm. Cytotoxicity was expressed as the concentration of drug inhibiting cell growth by 50% (IC_50_).

## 4. Conclusions

Phytochemical investigation of the rhizomes of *A. rivularis* var. *flore-minore*, led to the isolation of five new oleanane-type triterpenoid saponins **1**−**5** along with five known saponins **6**−**10**. Their structures were elucidated on the basis of spectroscopic studies and chemical evidence. These compounds were based on three types of aglycones, *i.e.*, gypsogenin, oleanolic acid and 21α-hydroxyoleanolic acid, and the third type of aglycone was found from the *Ranunculaceae* family for the first time. All of the compounds were tested for cytotoxicity against HL-60, HepG2, A549 and HeLa cell lines. The monodesmosidic saponins **6**−**8** exhibited cytotoxic activity toward all cancer cell lines, with IC_50_ values in the 7.25−22.38 μM range.

## Supplementary Materials

Physical and spectroscopic data of compounds **6**−**10**, along with NOESY and HMBC correlations for compounds **2**−**5** are available as supporting data, which can be accessed at: http://www.mdpi.com/1420-3049/19/2/2121/s1.
