# Supplementary material for: Cytotoxic Oleanane-Type Triterpenoid Saponins from the Rhizomes of Anemone rivularis var. flore-minore
Source: Molecules. 2014 Feb 18;19(2):2121–34. doi: 10.3390/molecules19022121 (PMC6270712; doi:10.3390/molecules19022121)

Supplementary Materials

Identification Data of Compounds 6–10

Compound 6: sapindoside B

White amorphous powder; $[\alpha]_D^{22}$ +10.0 (*c* 0.08, MeOH); ESI-MS (pos. ion mode) *m/z* 905 $[M+Na]^+$; ESI-MS (neg. ion mode) *m/z* 881 $[M-H]^-$, 917 $[M+Cl]^-$; ESI-MS/MS (neg. ion mode, parent ion at *m/z* 881) *m/z* 749 $[881-132]^-$, 603 $[749-146]^-$, 471 $[603-132]^-$. ^{1}H-NMR (500 MHz, pyridine-d_5) δ: 0.90, 0.91, 0.97, 0.99, 1.10, 1.22 (each 3H, s, CH_3), 1.53 (3H, d, *J* = 6.2 Hz, CH_3 of rha), 3.26 (1H, dd, *J* = 13.8, 4.0 Hz, H-18), 5.04 (1H, d, *J* = 6.7 Hz, H-1 of ara), 5.32 (1H, d, *J* = 7.6 Hz, H-1 of xyl), 5.44 (1H, br s, H-12), 6.31 (1H, br s, H-1 of rha); ^{13}C-NMR data, see Table S1.

Compound 7: pulsatilla saponin D

White amorphous powder; $[\alpha]_D^{22}$ +16.4 (*c* 0.11, MeOH); ESI-MS (pos. ion mode) *m/z* 935 $[M+Na]^+$; ESI-MS (neg. ion mode) *m/z* 911 $[M-H]^-$, 947 $[M+Cl]^-$; ESI-MS/MS (neg. ion mode, parent ion at *m/z* 911) *m/z* 765 $[911-146]^-$, 749 $[911-162]^-$, 603 $[749-146]^-$, 471 $[603-132]^-$. ^{1}H-NMR (500 MHz, pyridine-d_5) δ: 0.90, 0.91, 0.97, 0.99, 1.06, 1.20 (each 3H, s, CH_3), 1.63 (3H, d, *J* = 6.1 Hz, CH_3 of rha), 3.25 (1H, dd, *J* = 13.7, 3.4 Hz, H-18), 4.95 (1H, d, *J* = 6.8 Hz, H-1 of ara), 5.09 (1H, d, *J* = 7.9 Hz, H-1 of glc I), 5.44 (1H, br s, H-12), 6.24 (1H, br s, H-1 of rha) ; ^{13}C-NMR data, see Table S1.

Compound 8: 3*β*-O-{*β*-D-xylopyranosyl-(1→3)-*α*-L-rhamnopyranosyl-(1→4)-[*β*-D-glucopyranosyl-(1→4)]-*α*-L-arabinopyranosyl} oleanolic acid

White amorphous powder; $[\alpha]_D^{22}$ +14.4 (*c* 0.11, MeOH); ESI-MS (pos. ion mode) *m/z* 1051 $[M+Na]^+$; ESI-MS (neg. ion mode) *m/z* 1027 $[M-H]^-$; ^{1}H-NMR (500 MHz, pyridine-d_5) δ: 0.81, 0.94, 0.97, 0.99, 1.14, 1.29, 1.30 (each 3H, s, CH_3), 1.55 (3H, d, *J* = 6.2 Hz, CH_3 of Rha), 3.23 (1H, dd, *J* = 4.2, 13.9 Hz, H-3), 3.28 (1H, dd, *J* = 4.4, 11.8 Hz, H-18), 4.72 (1H, d, *J* = 7.1 Hz, H-1 of Ara), 5.10 (1H, d, *J* = 7.8 Hz, H-1 of Glc I), 5.34 (1H, d, *J* = 7.5 Hz, H-1 of Xyl), 5.44 (1H, br s, H-12), 6.32 (1H, s, H-1 of Rha); for ^{13}C-NMR spectroscopic data, see Table S1.

Compound 9: sieboldianoside B

White amorphous powder; $[\alpha]_D^{22}$ −25.5 (*c* 0.20, MeOH); ESI-MS (pos. ion mode) *m/z* 1359 $[M+Na]^+$; ESI-MS (neg. ion mode) *m/z* 1335 $[M-H]^-$; ^{1}H-NMR (500 MHz, pyridine-d_5) δ: 0.87 (6H, s, 2 × CH_3), 0.85, 1.06, 1.14, 1.23, 1.27 (each 3H, s, CH_3), 1.51 (3H, d, *J* = 6.1 Hz, CH_3 of 3-*O*-Rha), 1.67 (3H, d, *J* = 6.1 Hz, CH_3 of 28-*O*-Rha), 3.15 (1H, dd, *J* = 3.8, 13.2 Hz, H-18), 3.26 (1H, dd, *J* = 4.2, 11.7 Hz, H-3), 4.83 (1H, d, *J* = 7.0 Hz, H-1 of Ara), 4.97 (1H, d, *J* = 7.8 Hz, H-1 of 28-*O*-Glc III), 5.33 (1H, d, *J* = 7.8 Hz, H-1 of Xyl), 5.37 (1H, br s, H-12), 5.83 (1H, s, H-1 of 28-*O*- Rha), 6.22 (1H, d, *J* = 8.2 Hz, H-1 of 28-*O*-Glc II), 6.38 (1H, s, H-1 of 3-*O*-Rha); for ^{13}C-NMR spectroscopic data, see Table S1.

Compound 10: 3-*O*-α-L-arabinopyranosyl gypsogenin 28-*O*-α-L-rhamnopyranosyl-(1→4)-β-D-glucopyranosyl-(1→6)-β-D-glucopyranosyl ester

White amorphous powder; $[\alpha]_D^{22}$ +8.2 (*c* 0.25, MeOH); ESI-MS (pos. ion mode) *m/z* 1095 $[M+Na]^+$; ESI-MS (neg. ion mode) *m/z* 1071 $[M-H]^-$, 939 $[1071-132]^-$, 601 $[1071-146-162-162]^-$; ^{1}H-NMR (500 MHz, pyridine-d_5) δ: 0.85, 0.87, 0.89, 1.05, 1.21, 1.30 (each 3H, s, CH_3), 1.68 (3H, d, J = 6.1 Hz, CH_3 of Rha), 3.14 (1H, dd, J = 3.3, 13.4 Hz, H-18), 4.90 (1H, d, J = 7.0 Hz, H-1 of Ara), 4.97 (1H, d, J = 7.7 Hz, H-1 of Glc III), 5.39 (1H, br s, H-12), 5.82 (1H, s, H-1 of Rha), 6.21 (1H, d, J = 8.1 Hz, H-1 of Glc II); for ^{13}C-NMR spectroscopic data, see Table S1.

Table S1. ^{13}C-NMR (125 MHz) chemical shifts of saponins **6**–**10** in pyridine-d_5.

C	6	7	8	9	10	C	6	7	8	9	10
1	38.9	38.9	38.8	38.8	38.2	3	82.9	72.4	82.9	82.8	
2	26.3	26.2	26.7	26.6	25.4	4	72.9	74.1	72.9	72.9	
3	81.0	81.0	88.6	88.6	81.5	5	69.7	69.6	69.6	69.6	
4	43.5	43.4	39.5	39.5	55.4	6	18.4	18.6	18.5	18.4	
5	47.6	47.7	56.0	55.9	47.8	Xyl					
6	18.0	18.1	18.5	18.5	20.7	1	107.5		107.5	107.5	
7	32.8	32.8	33.1	33.1	32.5	2	75.1		75.7	75.6	
8	39.7	39.7	39.7	39.8	40.2	3	78.3		78.5	78.4	
9	48.1	48.1	48.0	48.0	48.1	4	71.0		71.1	71.1	
10	36.8	36.8	37.0	37.0	36.3	5	67.3		67.4	67.4	
11	23.6	23.6	23.7	23.7	23.4	Glc I					
12	122.5	122.5	122.4	122.8	122.5	1		106.7	106.6		
13	144.7	144.8	144.8	144.0	144.0	2		75.4	75.4		
14	42.1	42.1	42.1	42.1	42.2	3		78.5	78.4		
15	28.3	28.3	28.3	28.2	28.2	4		71.1	71.2		
16	23.8	23.8	23.7	23.3	23.5	5		78.7	78.8		
17	46.6	46.6	46.7	47.0	47.0	6		62.4	62.5		
18	41.9	41.9	42.0	41.6	41.6	28-*O*-sugar					
19	46.3	46.3	46.5	46.2	46.1	Glc II					
20	30.9	30.9	30.9	30.7	30.7	1				95.6	95.5
21	34.1	34.1	34.2	33.9	33.9	2				73.8	73.8
22	33.1	33.2	33.2	32.5	32.4	3				78.7	78.6
23	63.9	63.8	28.1	28.1	206.4	4				70.8	70.7
24	14.1	14.0	17.2	17.2	10.4	5				78.0	78.0
25	16.0	16.0	15.5	15.6	15.6	6				69.1	69.1
26	17.4	17.4	17.4	17.4	17.4	Glc III					
27	26.1	26.1	26.1	26.0	26.1	1				104.8	104.8
28	180.2	180.2	180.3	176.5	176.6	2				75.3	75.3
29	33.2	33.2	33.3	33.1	33.0	3				76.4	76.4
30	23.7	23.7	23.7	23.6	23.7	4				78.2	78.1
3-*O*-sugar						5				77.1	77.1
Ara						6				61.2	61.2

Table S1. *Cont.*

C	6	7	8	9	10	C	6	7	8	9	10
1	104.6	104.4	105.2	105.2	105.3	Rha II					
2	75.6	76.2	75.5	75.0	72.4	1				102.7	102.7
3	75.1	75.0	74.7	74.7	74.3	2				72.5	72.5
4	69.5	80.4	80.2	69.3	69.2	3				72.7	72.7
5	66.2	65.4	65.2	65.7	66.7	4				73.9	73.9
Rha I						5				70.2	70.2
1	101.3	101.6	101.4	101.3		6				18.5	18.5
2	71.9	72.2	71.8	71.9							

Figure S1. Key NOESY and HMBC correlations for compound **2**.

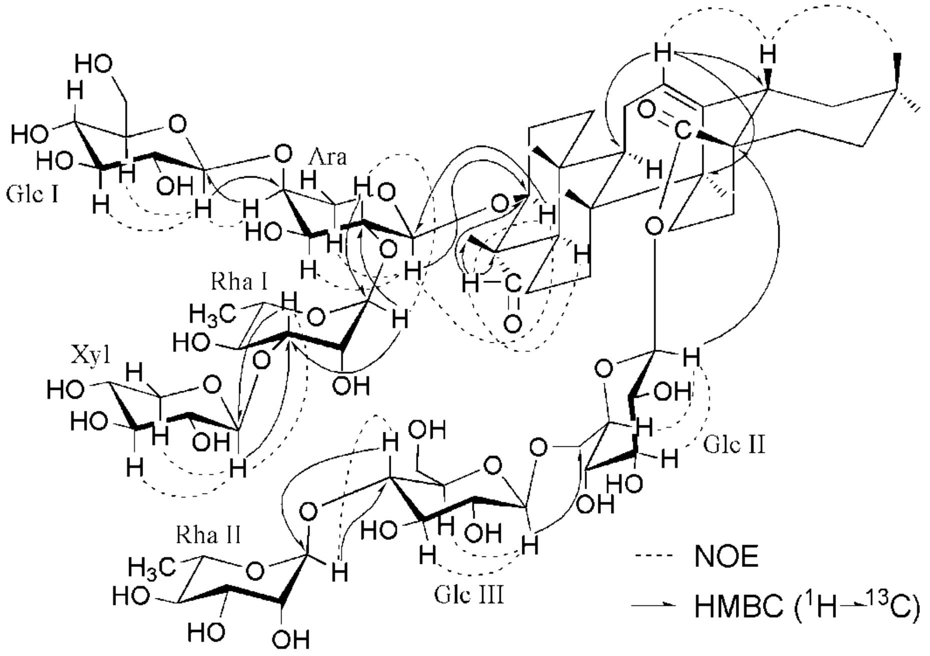

Figure S2. Key NOESY and HMBC correlations for compound **3**.

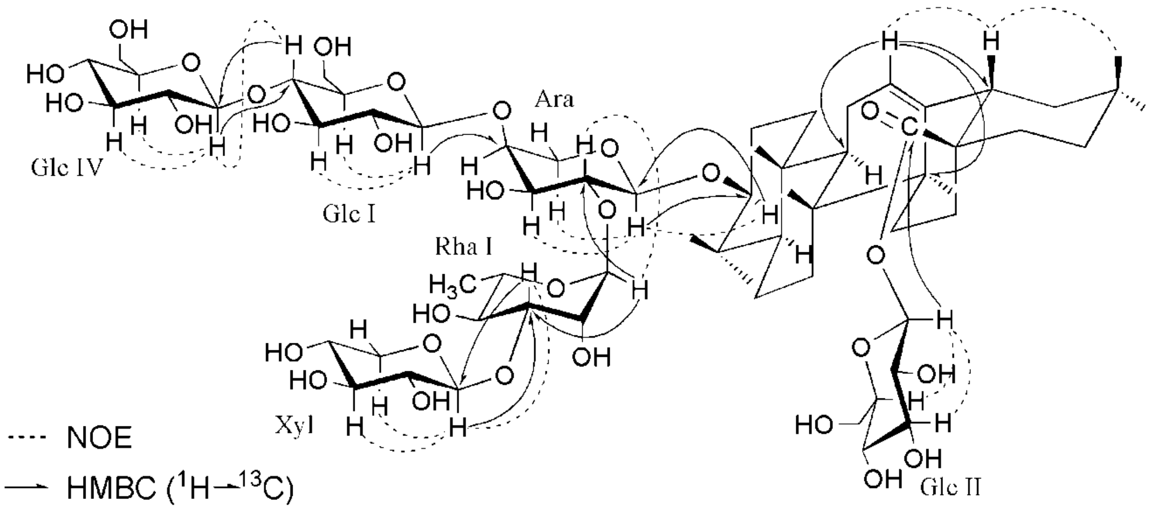

Figure S3. Key NOESY and HMBC correlations for compound **4**.

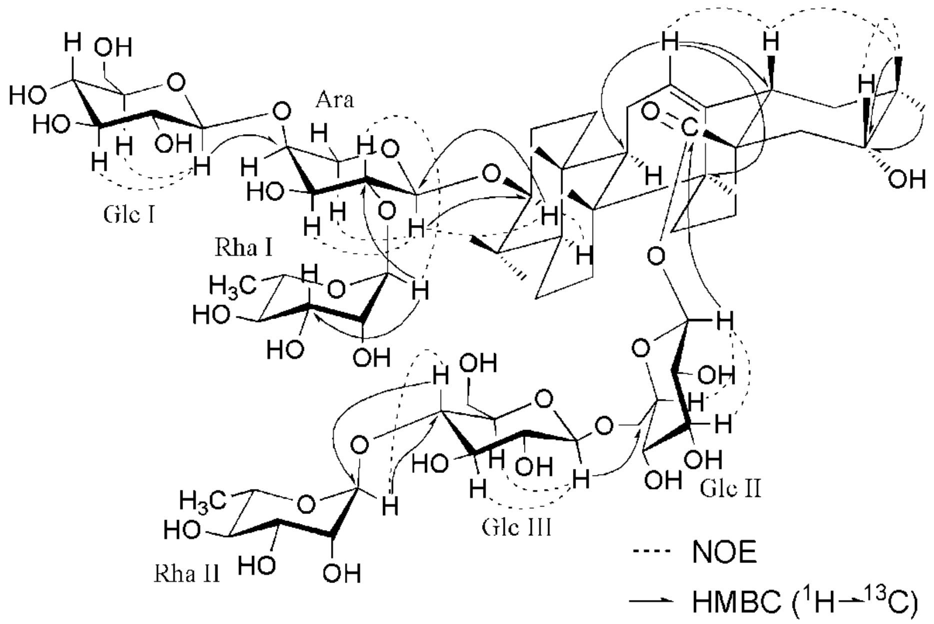

Figure S4. Key NOESY and HMBC correlations for compound **5**.

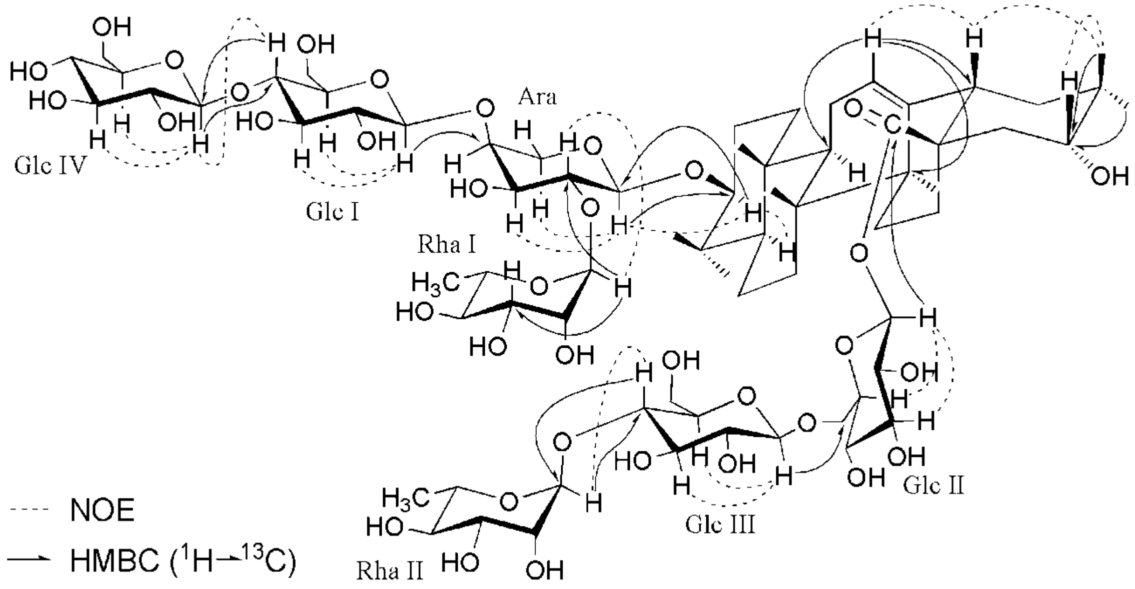

Supplement: Supplementary file 1 [file molecules-19-02121-s001.pdf]
